# Photoluminescence quenching of dye molecules near a resonant silicon nanoparticle

**DOI:** 10.1038/s41598-018-24492-y

**Published:** 2018-04-17

**Authors:** Mikhail V. Zyuzin, Denis G. Baranov, Alberto Escudero, Indranath Chakraborty, Anton Tsypkin, Elena V. Ushakova, Florain Kraus, Wolfgang J. Parak, Sergey V. Makarov

**Affiliations:** 1 Fachbereich Physik, Philipps-Universität Marburg, Renthof 7, 35037 Marburg, Germany; 2 Department of Physics, Chalmers University of Technology, 412 96 Gothenburg, Sweden; 3 Department of Nanophotonics and Metamaterials, ITMO University, St. Petersburg, 197101 Russia; 4 Moscow Institute of Physics and Technology, Dolgoprudny, 141700 Russia; 5 Instituto de Ciencia de Materiales de Sevilla, CSIC – Universidad de Sevilla, Calle Américo Vespucio 49, E-41092 Seville, Spain; 6 Fachbereich Physik und Chemie und CHyN, Universität Hamburg, Luruper Chausee 149, 22607 Hamburg, Germany; 7 Fachbereich Chemie, Philipps-Universität Marburg, Hans-Meerwein-Straße 4, 35032 Marburg, Germany

## Abstract

Luminescent molecules attached to resonant colloidal particles are an important tool to study light-matter interaction. A traditional approach to enhance the photoluminescence intensity of the luminescent molecules in such conjugates is to incorporate spacer-coated plasmonic nanoantennas, where the spacer prevents intense non-radiative decay of the luminescent molecules. Here, we explore the capabilities of an alternative platform for photoluminescence enhancement, which is based on low-loss Mie-resonant colloidal silicon particles. We demonstrate that resonant silicon particles of spherical shape are more efficient for photoluminescence enhancement than their plasmonic counterparts in spacer-free configuration. Our theoretical calculations show that significant enhancement originates from larger quantum yields supported by silicon particles and their resonant features. Our results prove the potential of high-index dielectric particles for spacer-free enhancement of photoluminescence, which potentially could be a future platform for bioimaging and nanolasers.

## Introduction

Resonant enhancement of photoluminescence (PL) from dye molecules is of great importance for bioimaging^1,2^ and nanolasers^3^. Traditionally, efforts to increase the PL signal from luminescent probes have relied on the use of plasmonic nanoantennas (made out of gold or silver), which enable an enhancement of the near fields around the particles and, therefore, increase the excitation rate of the dye molecules^4–6^. The major challenge of using plasmonic particles is related to the intense non-radiative decay of luminescent sources, referred to as quenching, originating from large Joule damping when a dye molecule is attached close to a particle surface^4,7–10^. Although a number of alternative materials for plasmonics, such as doped semiconductors, have been suggested^11^, this effect presents a severe problem in nanophotonics hindering the realization of nanolasers^12^ and single-photon sources^13^. This drawback of the dramatic quenching in plasmon enhanced PL can be solved via incorporation of a few nanometers thick spacer between the molecules and the particle surface, which increases the quantum yield and PL intensity^6,14^. However, this approach requires an additional technological step for coating of particles with a spacer.

On the other hand, there is an alternative route towards enhanced PL offered by high-index dielectric nanoantenas^15^. Subwavelength particles made of these materials, such as silicon, exhibit resonant features in the visible range associated with their electric and magnetic dipole Mie modes along with low level of Joule loss^16^. A number of coupled nanoparticle-molecules implementations where the nanoresonators were covered by emitters^17,18^, placed on emitting material^19^, or emitters were incorporated inside the resonant nanoparticles^20^ have been recently demonstrated. The resonant behaviour of such particles^21,22^ provides a natural way for acceleration of spontaneous emission and PL enhancement^23–28^. However, performances of plasmonic and high-index dielectric particles in terms of PL quenching and related enhancement have not been compared directly in previous experiments. Moreover, experimental study of PL dependence of an emitting dipole on the distance from the dielectric particle surface is still lacking, which could be useful for optimization of biomarkers or nanolasers based on the silicon particles.

In this paper, for the first time to the best of our knowledge, we provide an experimental study of quenching of dye molecules placed around silicon nanoparticles. We carry out both experimental and theoretical comparative analysis of the dependencies of the photoluminescence intensity and the decay lifetimes for silicon (Si) and gold (Au) single spherical particles on the distance between the molecules and particles surface. We compare them with reference yttrium vanadate (YVO_4_) particles coated by the same dye, which are low-index dielectric objects without any resonances and losses in the visible range. The results indicate that Si particles exhibit the most favourable properties for PL enhancement at a few nanometers distance between molecules and the particle, when the plasmonic antennas fail to provide PL enhancement due to quenching.

## Theoretical framework

**Figure 1:**
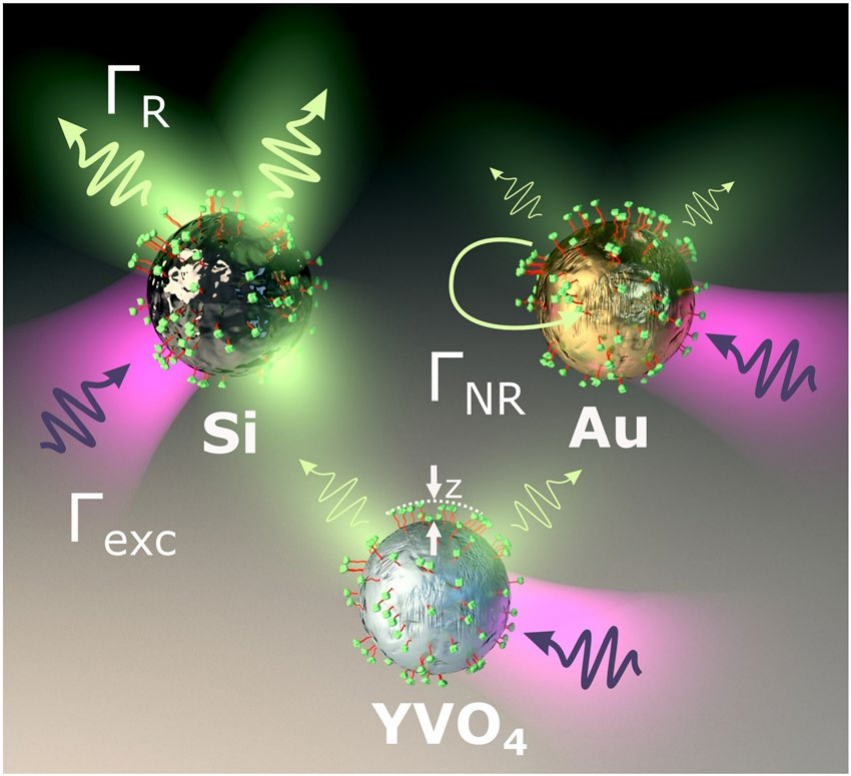
Schematic illustration of PL mediated by plasmonic and dielectric antennas: spontaneous emission (radiative channel, ${{\rm{\Gamma }}}_{R}$) of a dye molecule attached to an Au nanoantenna (with the distance z to the Au surface) is suppressed by non-radiative losses (${{\rm{\Gamma }}}_{NR}$), while a Si particle enables radiative emission enhancement along with low non-radiative losses. A low-index (YVO_4_) particle has no near-field enhancement and negligible ${{\rm{\Gamma }}}_{NR}$.

To start with, let us briefly recall the basic picture of the PL enhancement by optical nanoantennas. The electromagnetic enhancement of PL is schematically illustrated in Fig. 1. A pump laser at frequency ${\omega }_{exc}$ excites fluorescent molecules attached to a nanoantenna at a rate ${{\rm{\Gamma }}}_{exc}$, which spontaneously emit radiation with frequency ${\omega }_{em}$ at a rate ${{\rm{\Gamma }}}_{R}$. Part of the energy is also dissipated non-radiatively at a rate ${{\rm{\Gamma }}}_{NR}$. The whole structure is embedded in a lossless host medium with permittivity ${\varepsilon }_{h}$. In the following, we will consider three different nanoantenna materials: Au for plasmonic antennas, Si for resonant high-index dielectric antennas, and YVO_4_ as a reference non-resonant dielectric particle with low refractive index ($n\approx 2$). In all cases the nanoantennas are assumed to be immersed in water.

PL is a two-step process, whose intensity is dictated by two crucial factors: (i) efficient excitation rate ${{\rm{\Gamma }}}_{exc}$ of the dye molecules by pump laser, and (ii) large total quantum yield $\eta$ at the emission frequency^4,29^. The resulting PL intensity is proportional to the product of both: ${I}_{PL}\propto \eta {{\rm{\Gamma }}}_{exc}$.

The excitation rate of a molecule is defined according to ${{\rm{\Gamma }}}_{exc}\propto {|{{\bf{p}}}_{0}{{\bf{E}}}_{exc}|}^{2}$ with ${{\bf{p}}}_{0}$ being the molecule’s transition dipole moment and ${{\bf{E}}}_{exc}$ the local electric field at the excitation wavelength. Therefore, it can be increased via placing dye molecules in the vicinity of a nanoantenna hotspot, where intense electric fields at the excitation wavelength are generated. For spherical geometry, the local excitation field experienced by a molecule is given by^4^

1 $${{\bf{E}}}_{exc}={{\bf{E}}}_{0}+{k}_{exc}^{2}\hat{{\bf{G}}}({{\bf{r}}}_{m},{{\bf{r}}}_{NP};{\omega }_{exc}){\bf{p}},$$

where $\hat{{\bf{G}}}({{\bf{r}}}_{m},{{\bf{r}}}_{NP};{\omega }_{exc})$ is the electric Green tensor, ${k}_{exc}={\omega }_{exc}/c$, ${{\bf{r}}}_{m}\,$and ${{\bf{r}}}_{NP}$ denote the position of molecule and nanoparticle, respectively, and ${\bf{p}}$ is the nanoparticle electric dipole moment induced by the excitation field ${{\bf{E}}}_{0}$.

The total quantum yield $\eta$ of the emitter is determined by the competition between far-field radiation, Joule losses, and internal non-radiative losses which are accounted for by the intrinsic quantum yield ${\eta }_{0}$ (ref.^30^):

2 $$\eta =\frac{{{\rm{\Gamma }}}_{R}}{{{\rm{\Gamma }}}_{R}+{{\rm{\Gamma }}}_{NR}+{{\rm{\Gamma }}}_{0}(1-{\eta }_{0})/{\eta }_{0}},$$

where ${{\rm{\Gamma }}}_{0}$ and ${{\rm{\Gamma }}}_{R}$ are the radiative decay rates in free space and near a nanoantenna, respectively.

The radiative decay rate from a subwavelength nanoantenna is dominated by the electric and magnetic dipole contributions and is estimated as^29^:

3 $${{\rm{\Gamma }}}_{R}\propto {n}_{h}{|{{\bf{p}}}_{0}+{\alpha }_{e}{k}_{em}^{2}\hat{{\bf{G}}}({{\bf{r}}}_{m},{{\bf{r}}}_{NP};{\omega }_{em}){{\bf{p}}}_{0}|}^{2}+{n}_{h}^{3}{|i{\alpha }_{m}{k}_{em}{\rm{\nabla }}\times \hat{{\bf{G}}}({{\bf{r}}}_{m},{{\bf{r}}}_{NP};{\omega }_{em}){{\bf{p}}}_{0}|}^{2},$$

where ${k}_{em}={\omega }_{em}/c$, and ${\alpha }_{e}$ and ${\alpha }_{m}$ are the electric and magnetic dipole polarizabilities of the nanoparticle, respectively (see *Supporting Information*). In Eq. (3) the first term represents the electric dipolar (ED) radiation, while the second term represents the power emitted by magnetic dipole (MD) induced in the particle. To account for various orientation of the molecules with respect to the excitation field, we assumed that molecules are oriented transversely or longitudinally with probabilities of 1/3 and 2/3, respectively (see *Supporting Information*). Note that the magnetic contribution to the radiative rate is negligible for plasmonic nanoantennas.

Finally, the non-radiative decay rate of a molecule positioned in the vicinity of a nanosphere can be estimated with the quasistatic theory of quenching^31,32^, which treats the spherical particle as a plane surface. In this picture, the non-radiative decay rate of a molecule at a distance $z$ from the particle surface takes the form:

4 $${{\rm{\Gamma }}}_{NR}(z)=\frac{1}{4}{\rm{Im}}\frac{\varepsilon -{\varepsilon }_{h}}{\varepsilon +{\varepsilon }_{h}}\frac{1}{{k}_{h}^{3}{z}^{3}},$$

with ${\varepsilon }_{h}$ and ${k}_{h}$ being the permittivity and wavenumber at the emission frequency in the host medium (water), respectively, and $\varepsilon$ the permittivity of the nanoparticle material. From Eqs. (1–4) one can easily obtain the particle-free PL intensity ${I}_{PL}^{0}$ by equating ${\alpha }_{e}$, ${\alpha }_{m}$, and ${{\rm{\Gamma }}}_{NR}$ to zero.

**Figure 2:**
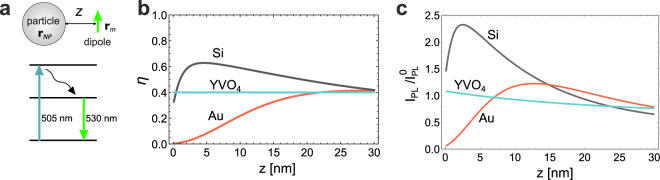
(**a**) Schematic illustration of the simulated system and the energy structure of a DY-505 dye molecule. (**b**) The total quantum yield of a dye molecule given by Eq. (2) as a function of the distance *z* between the molecule and the particle surface for Si, Au and YVO_4_ particles in water. (**c**) Calculated PL intensity ${I}_{PL}\propto \eta {\Gamma }_{exc}$ from 150 nm Au, Si and YVO_4_ spherical particles in water. The PL values are normalized by the respective value of PL intensity in water without the nanoparticle.

The results predicted by this model are presented in Fig. 2 for Au, Si and YVO_4_ particles of 150 nm diameter embedded in water, and a Rhodamine 110 based dye molecule (DY-505) linked to the particle. The DY-505 fluorophore with an excitation wavelength of 505 nm and an emission centred at 530 nm is assumed as the luminescent material, Fig. 2(a). The intrinsic quantum yield of the molecule is taken to be ${\eta }_{0}=0.4$ (ref.^26^), and the natural lifetime of DY-505 fluorophore is estimated as 4 ns (refs^33,34^).

The significant difference in the behaviour of the quantum yield of a dye attached to plasmonic and dielectric particles is illustrated in Fig. 2(b) as a function of distance *z*. This magnitude strongly depends on both the particle material and the distance *z* between the dye and the particle surface. At distances below 10 nm the quantum yield of a dye molecule attached to an Au particle is greatly reduced due to large non-radiative losses. At the same time, dielectric Si and YVO_4_ particles allow for much larger values of quantum yield, even at small distances. Notably, the quantum yield from an YVO_4_ particle is fixed at the “vacuum” value of 0.4, because of the negligible imaginary part of its dielectric function (thus negligible non-radiative losses ${{\rm{\Gamma }}}_{NR}$), whereas the quantum yield of the dye attached to a Si particle demonstrates non-monotonic dependence. It is also seen that the total quantum yield $\eta$ in the case of Si particles may exceed the vacuum value ${\eta }_{0}$ due to Purcell enhancement of the spontaneous emission rate ${{\rm{\Gamma }}}_{R}$, as Eq. (2) suggests. This difference in the quantum yield behaviour mostly determines the dependencies presented in Fig. 2(c), where we show the PL intensities for all three types of particles normalized by the respective particle-free value ${I}_{PL}^{0}$.

## Results and Discussion

**Figure 3:**
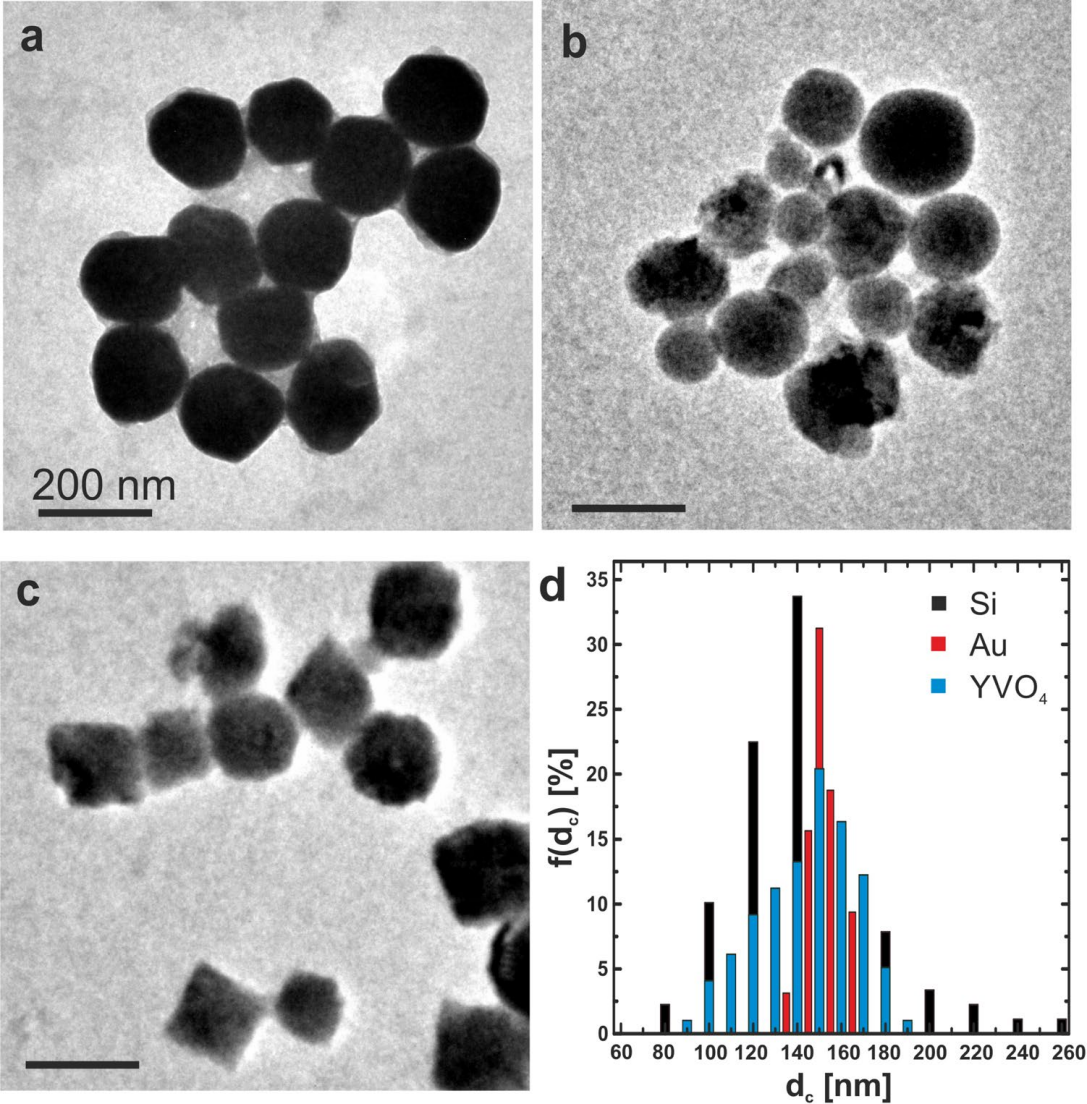
TEM images of (**a**) gold, (**b**) silicon and (**c**) YVO_4_ particles as created, without coating with integrated DY-505. (**d**) Size distributions f(d_c_) of the corresponding particles. All scale bars correspond to 200 nm.

Si and YVO_4_ particles were fabricated by laser ablation and synthesized, respectively, in aqueous phase with an average diameter ± standard variation of the inorganic core d_c_(Si) = 140 ± 47 nm and d_c_(YVO_4_) = 145 ± 25 nm, as determined by transmission electron microscopy (TEM, Fig. 3)^35,36^. Gold particles in aqueous phase were commercially purchased with an average diameter d_c_(Au) = 150 ± 12 nm. More details on creation of nanoparticles are given in the *Supporting Information*. According to previous measurements, the fabricated Si particles have a crystalline structure^37,38^, as well as the YVO_4_ particles^39,40^. The hydrodynamic diameters of the particles were d_h_(Si) = 170 ± 16 nm, d_h_(Au) = 150 ± 5 nm, d_h_(YVO_4_) = 150 ± 7 nm, as determined in Milli-Q water with dynamic light scattering (DLS, see Table SI in the *Supporting Information*). Error bars corresponds to the standard deviation from 3 measurements.

**Figure 4:**
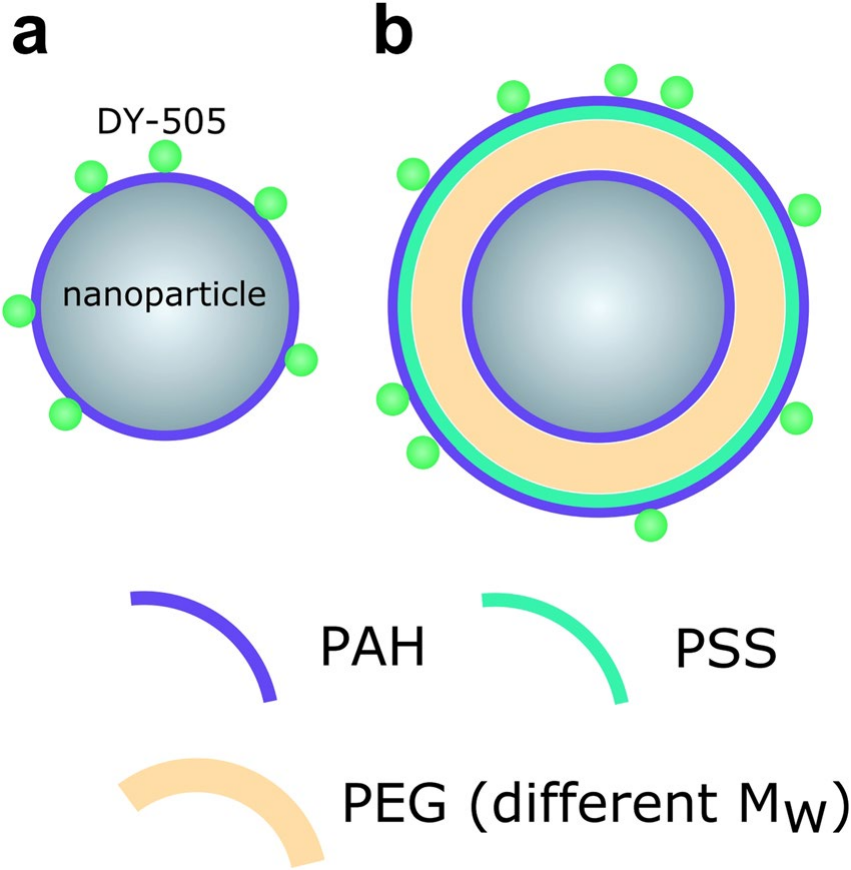
Schematic illustration of coated particles, (**a**) with no spacer in geometry particle core/PAH-DY-505, (**b**) with PEG spacer in geometry particle core/PAH/PSS/PAH-DY-505.

In order to demonstrate the feasibility of the proposed approach, the fabricated Si, Au, and YVO_4_ particles were coated with a fluorescent Rhodamine 110 based dye (DY-505), which was situated at different distances from the particle surface. For the closest distance, the fluorophore was added to the particles with a DY-505 modified PAH layer. For increasing the distance, first a layer of PAH was added to the particle surface^41^, on which then a layer of polyethylene glycol (PEG) of different molecular weights was added. The higher the molecular weight of the PEG, the bigger the hydrodynamic diameter^42,43^. The spacer layer of PEG was then capped with one layer of poly(sodium 4-styrenesulfonate) (PSS). Finally, a last layer with poly(allylamine hydrochloride) (PAH) with incorporated DY-505 dye was added (Fig. 4). We studied a set of 3 × 5 = 15 samples, in which each particle (3 different cores: Si, Au, YVO_4_) was coated with PEG of different molecular weights (4 different M_w_ from 2 to 20 kDa) except one coating without PEG in which the core was directly coated by PAH-DY-505 as shown in Fig. 4a.

The basic physicochemical properties (hydrodynamic diameters d_h_, zeta potentials ξ) as recorded in Milli-Q water are shown for all coated particles in the *Supporting Information*. The successive coatings of the studied particles with PEG and the different polyelectrolytes (PSS, PAH-DY-505) was hereby evidenced by the changes in the zeta-potential of the particle suspensions. All coated particles possess positive zeta potential, indicating the successful final attachment of cationic PAH conjugated with DY-505. Moreover, no significant agglomeration was observed, especially for the Au and Si particles, as indicated by the DLS measurements (data are presented in the *Supporting Information*). Distances between the dye and the particle surface were controlled by the thickness of the PEG shell. Values were estimated from the work of del Pino *et al*.^43^, see the *Supporting Information*.Based on these results, the total thickness z of the PEG spacer layer varied from 5 to 25 nm.

**Figure 5:**
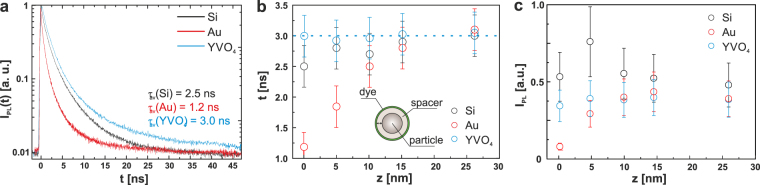
(**a**) Measured time-resolved normalized PL I_PL_(t) from coated Si, Au, and YVO_4_ particles without PEG spacers. (**b**) Measured values of PL lifetime τ for dyes in the PAH shell around Si, Au, and YVO_4_ particles versus the distance z between the particle surface and the dye molecules. (**c**) Measured PL intensity from Si, Au, and YVO_4_ particles versus spacer thickness for the same particle concentration.

To illustrate the quenching effect by different types of the dye-coated particles, we first present the lifetime values extracted from time-resolved PL measurements, Fig. 5(a,b). In order to study PL decay experimentally, we applied 70 ps laser pulses at wavelength of 405 nm to the dye-coated particles deposited on a glass substrate using a laser scanning confocal microscope MicroTime 100 (PicoQuant) (for details, see the *Supporting Information*). The results clearly demonstrate non-exponential decay character for all three types of particles, which is common for such molecules and may be attributed to high sensitivity to surrounding conditions^34^. Fig. 5(b) shows a prominent difference in the averaged PL lifetime values of dye molecules attached to Si (${\tau }_{av}=2.5$ ns), Au (${\tau }_{av}=1.2$ ns), and the reference YVO_4_ (${\tau }_{av}=3.0$ ns) particles without spacers (i.e. the samples with no PEG layer); with previously reported value of 4.1 ns for averaged decay time in water^33^. The relatively fast spontaneous emission of the dye molecules attached to Au particles is attributed to an increased non-radiative recombination originating from the excitation of high-order modes in Au particles. In turn, the relaxation rate of this dye attached to Si particles exhibits a moderate enhancement originating from the Purcell effect. YVO_4_ particles show the largest lifetime values almost independent of the spacer thickness, which is consistent with the non-resonant response of these particles and excludes influence of spacers on quenching (see the *Supporting Information*). The observed notable increase of the PL lifetime at larger spacer thickness for Au and Si particles is in agreement with the theoretical predictions.

Steady-state PL measurements in a commercial fluorimeter (Fluorolog-3, Horiba Jobin Yvon) were performed for the same amount of nanoparticles in each case using excitation at the wavelength of 505 nm (for details, see the *Supporting Information*). Since the surface modification of the different nanoparticles was carried out for nanoparticles with similar initial size and ζ potential, with the same amount of PEG spacer and always in a great excess of both polymers or dye conjugated polymer, it is a good approximation to consider that the final samples show a very similar dye/nanoparticle ratio, and thus the experimental data can reasonably be compared with the outcome of our model. PL intensity measurements at an emission wavelength of 530 nm from the spacer-free particles, as shown in Fig. 5(c), indicate that the dye-coated Si particles exhibit the highest PL signal, originating from both the Purcell effect and the near-field enhancement of the excitation rate, as predicted by our calculations (Fig. 2). Remarkably, the PL enhancement with the Si particles is clearly observable despite their broader size distribution relatively to the Au and YVO_4_ particles, which only increases variance of the measured values.

In order to prevent the harmful effect of quenching on the PL intensity, spacers were incorporated in the design of particles. This increases the distance z between the dye molecules and the particle surface, thus reducing the non-radiative decay ${{\rm{\Gamma }}}_{NR}$ and increasing the total quantum yield $\eta$. As the PEG spacer layer gets thicker, the coating also reduces the local field enhancement at the excitation wavelength. This results in a local maximum of PL signal at z = 5 nm for Si and 15 nm for Au particles, Fig. 5(c). With further thickness increase, the PL signal from Si particles gradually decreases, until it becomes comparable to the PL signal from Au and YVO_4_ particles, when the quenching effect becomes less pronounced. Hence, the dependencies of the PL intensity of the spacer thickness allow for optimizing the particle dimensions to get the best performance.

The absence of strong quenching and high electric field enhancement make Si particles, supporting Mie resonances in the visible range, promising nanoantennas with potential applications in bioimaging. As with other particles^44,45^, dye coated Si particles are readily incorporated by cells (data are given in the *Supporting Information*). Also after internalization, Si particles coated by the DY-505 dye without a spacer had higher PL signal than similar Au particles.

## Conclusion

To conclude, we studied experimentally and theoretically the quenching effect for DY-505 dye molecules attached to different inorganic particles, whereby the distances of the dyes from the particle surfaces were varied. We demonstrated that the attachment of dye to silicon resonant particles may enhance the photoluminescence of the dye up to 3 times and 2 times as compared to that of attachment to plasmonic (gold) and non-resonant dielectric (YVO_4_) particles, respectively. Such enhancement is due to a strong domination of the radiative losses over the non-radiative ones in silicon particles supporting Mie resonances, whereas the non-radiative losses are too high for resonant gold particles, and YVO_4_ does not support any resonances or optical field enhancement. Our findings point out to the potential of resonant high-index nanoparticles for the development of bioimaging techniques.

## Electronic supplementary material

Supporting Information
