# Supplementary material for: Photoluminescence quenching of dye molecules near a resonant silicon nanoparticle
Source: Sci Rep. 2018 Apr 17;8:6107. doi: 10.1038/s41598-018-24492-y (PMC5904138; doi:10.1038/s41598-018-24492-y)
Supplement: Supplementary file 1 — Supporting Information [file 41598_2018_24492_MOESM1_ESM.pdf]

Supporting Information

Photoluminescence quenching of dye molecules around a resonant silicon nanoparticle

Mikhail V. Zyuzin,[†,¶] Denis G. Baranov,[*,‡,¶,§] Alberto Escudero,[†,♦]Indranath Chakraborty,[†] Anton Tsypkin,[¶] Elena V. Ushakova,[¶]Florain Kraus,[¥] Wolfgang J. Parak[†,#] and Sergey V. Makarov,[*,¶]

[†]Fachbereich Physik, Philipps-Universität Marburg, Renthof 7, 35037 Marburg, Germany

[‡]Department of Physics, Chalmers University of Technology, 412 96 Gothenburg, Sweden

[¶]ITMO University, St. Petersburg, Russia

[§]Moscow Institute of Physics and Technology, Dolgoprudny 141700, Russia

♦ Instituto de Ciencia de Materiales de Sevilla. CSIC – Universidad de Sevilla. CalleAméricoVespucio 49. E-41092 Seville, Spain.

[#]FachbereichPhysik und Chemie und CHyN, Universität Hamburg, LuruperChausee 149, 22607 Hamburg, Germany

[¥]FachbereichChemie, Philipps-Universität Marburg, Hans-Meerwein-Straße 4, 35032 Marburg, Germany.

[*]E-mail: denisb@chamers.se; s.makarov@metalab.ifmo.ru

Content:

1. Theoretical modelling
2. Materials
3. Synthesis of particles
4. Characterisation of as synthesized particles
5. Conjugation of polymer with dye
6. Polymer coating of particles
7. Basic colloidal characterization of coated particles
8. Photoluminescence studies
9. Time-resolved fluorescent microscopy
10. Cell studies
11. References

1. Theoretical modelling

The excitation rate and total quantum yield of dye molecules attached to various particles were calculated within the framework of the coupled dipole approximation.[1] Au particles were modelled as point electric dipoles with a dipolar polarizability α_e, while the dielectric particles were treated as a combination of electric and magnetic point dipoles with polarizabilities α_e and α_m, respectively. The polarizabilities are derived from Mie theory according to:[1]

$$\alpha_e = i\frac{3\varepsilon_h a_1}{2k_h^3}, \alpha_m = i\frac{3b_1}{2k_h^3} \qquad \text{(S1)}$$

with $\varepsilon_h = n_h^2$ being the host medium permittivity, $k_h = n_h\omega / c$ the wavenumber in the host medium, and a_1 and b_1 being the standard Mie coefficients.[2] Absolute values of the resulting polarizabilities for Au, Si, and YVO_4 particles in water are shown in Fig. S1 for the experimentally relevant diameters. An incident pump wave was assumed to induce linearly polarized electric and magnetic (for dielectric particles) dipole moments.

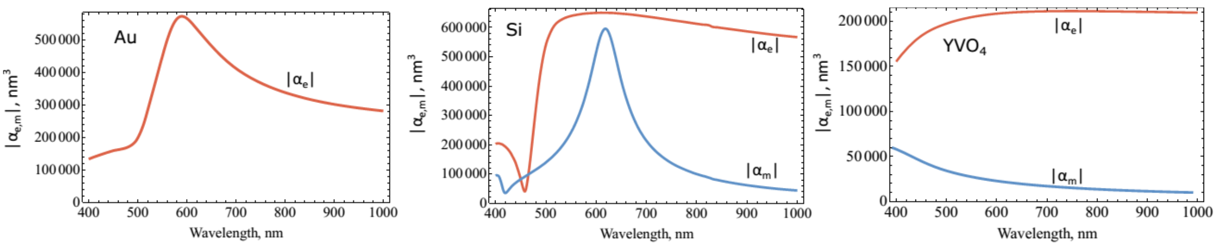

Fig. S1: Absolute values of the electric and magnetic polarizabilities of Au, Si, and YVO_4 particles for the experimentally relevant diameters.

Dye molecules were assumed to be uniformly distributed over the particle surface and randomly oriented. Depending on the molecule position and orientation with respect to the induced dipole moments in the particle, different values of the excitation rate may be achieved. To account for this disorder and simplify averaging, we assumed that molecules are oriented transversely or longitudinally with probabilities of 1/3 and 2/3, respectively.

Radiative decay of molecules originates from the electric and magnetic dipole radiation at the emission wavelength. Notably, the radiation rate is independent of the excitation polarization, which makes averaging easier. Neglecting the cooperative effects on the emission, we calculate independently the emission rate for each molecule orientation.

The common way to estimate the radiative decay rate is to calculate the imaginary part of the Green tensor at the position of the emitter.[3, 4] However, due to subwavelength dimension of the system, it is easier to compute the total electric $\mathbf{p}_{\text{tot}}$ and magnetic $\mathbf{m}_{\text{tot}}$ dipole moments of the structure and to express the decay rate as $\Gamma \sim n_h \left|\mathbf{p}_{\text{tot}}\right|^2 + n_h^3 \left|\mathbf{m}_{\text{tot}}\right|^2$ (see Ref. 3). The interference term here is absent since the radiated fields of the electric and magnetic dipoles are orthogonal. Accounting for different probabilities of transverse and longitudinal molecule orientation, we write the electric dipole contribution as

$$\begin{aligned}\Gamma_{\text{ED}}^{\perp} &\sim \frac{n_h}{3}\left|\mathbf{p}_0 + \alpha_e k_{\text{em}}^2 g_{\text{ED}}^{\perp}\left(z;\omega_{\text{em}}\right)\mathbf{p}_0\right|^2, \\ \Gamma_{\text{ED}}^{\parallel} &\sim \frac{2n_h}{3}\left|\mathbf{p}_0 + \alpha_e k_{\text{em}}^2 g_{\text{ED}}^{\parallel}\left(z;\omega_{\text{em}}\right)\mathbf{p}_0\right|^2,\end{aligned} \tag{S2}$$

where z is the distance between the molecule and the particle surface, $k_{\text{em}} = \omega_{\text{em}}/c$, $\mathbf{p}_0$ is the molecule transition dipole moment, and $g_{\text{ED}}^{\perp}$ and $g_{\text{ED}}^{\parallel}$ are the Green tensor elements given by

$$\begin{aligned}g_{\text{ED}}^{\perp}(z) &= \left(1 + \frac{i}{n_h k_{\text{em}} z} - \frac{1}{(n_h k_{\text{em}} z)^2}\right)\frac{e^{i n_h k_{\text{em}} z}}{z}, \\ g_{\text{ED}}^{\parallel}(z) &= \left(-\frac{2i}{n_h k_{\text{em}} z} + \frac{2}{(n_h k_{\text{em}} z)^2}\right)\frac{e^{i n_h k_{\text{em}} z}}{z}.\end{aligned} \tag{S3}$$

In the above equations, || and $\perp$ denote orientation of the molecule’s dipole moment parallel and transverse to the particle surface, respectively. Only electric dipoles with transverse orientation couple to the magnetic dipole mode of the particle. Therefore, the contribution of the magnetic dipole into the total radiative rate is given by

$$\Gamma_{\text{MD}}^{\perp} \sim \frac{2n_h^3}{3}\left|\alpha_m k_{\text{em}}^2 g_{\text{MD}}^{\perp}\left(z;\omega_{\text{em}}\right)\mathbf{p}_0\right|^2, \tag{S4}$$

with the corresponding magnetic Green tensor matrix element

$$g_{\text{MD}}^{\perp}(z) = \left(i - \frac{1}{n_h k z}\right)\frac{e^{n_h k z}}{z}. \tag{S5}$$

Note that Eq. (S4) contains only the particle’s magnetic dipole moment, since the molecule’s transition moment is purely electric.

2. Materials

Yttrium (III) acetylacetonate hydrate ($Y(C_5H_7O_2)_3 \times H_2O$, Sigma-Aldrich, 99.95%, #438790), sodium orthovanadate (Na_3VO_4, Aldrich, 99.98%, #450243), ethylene glycol (EG, Fluka, >99.5%, #03750), gold particles (Au, BBI solutions, #EM.GC150), Poly(allylamine hydrochloride) (PAH, 15 kDa, Sigma, #283215), dimethyl sulfoxide (DMSO, Sigma, #276855), DY-505-NHS-ESTER (DY-505, Dyomics, #505-01), borate buffer 50 mM at pH 8.5 (prepared from H_3BO_3 pH 8.5, Roth, #5614.1), sodium chloride (NaCl, Sigma, #S9888), α,ω-Bis-NHS ester-polyethyleneglycol (PEG-(NHCO-C_2H_4-CONHS)$_2$, 2000, 6000, 10000, 20000 Da, Rapp Polymere, #112000-35, #116000-35, #1110000-35, #1120000-35),poly(sodium 4-styrenesulfonate) (PSS, 70 kDa, Sigma-Aldrich, #243051), Dulbecco's Modified Eagle Medium (DMEM, Gibco, #11965084), penicillin and streptomicyn (P/S, Gibco, #15140122), L-glutamine (Gibco, #35050061), fetal bovine serum (FBS, Biochrom, #S0615), phosphate buffered saline (PBS, Biochrom, #L1825), cell mask orange (ThermoFisher, #C10045), hoechst 33342 (ThermoFisher, #62249), 8-well plate (Ibidi, #80826) were used. Miili-Q water (ddH_2O, Milli-Q Academic, Millipore, Billerica, USA) with a resistance greater than 18.2 MΩ $cm^{-1}$was used for all experiments.

3. Synthesis of particles

Fabrication of Si particles

Si particles were fabricated by laser ablation. A commercial femtosecond laser system (Femtosecond Oscillator TiF- 100F, AvestaPoject) was used, providing laser pulses at 800 nm central wavelength, with maximum pulse energies of up to 5 mJ, and pulse duration of 40 fs at the repetition rate of 1 kHz. The laser energy was varied and controlled by an acousto-optical modulator (R23080-3-LTD, Gooch and Housego) and a power meter (FielfMax II, Coherent), respectively, while the pulse duration was measured by an autocorrelator (AvestaPoject). Laser pulses were focused by a lens with a focal length of 5 cm on a Si 500-thick wafer covered by 2 mm water layer. The near-threshold regime of ablation was chosen to provide larger particles formation.[5] Concentration of synthesized samples was measured with inductively coupled plasma mass spectrometry (ICP-MS) as described in §7.

Synthesis of YVO_4 particles

YVO_4 particles were synthesised by wet chemistry from yttrium (III) acetylacetonate hydrate and sodium orthovanadate in a mixture of ethylene glycol (EG) – water at 120 ºC, as previously reported in the literature.[6, 7] Briefly, 0.1 mmol of yttrium acetylacetonate were dissolved in 2.5 mL of EG. The solution was mildly heated (75°C) under magnetic stirring to facilitate the dissolution for 1 hour. At the same time, 0.5 mmol of Na_3VO_4 were dissolved in a mixture of 1.5 mL of water and 1 mL of EG. After cooling down to room temperature, both solutions were mixed. The final EG:H_2O volumetric ratio was thus 3.5:1.5, and the $Y(C_5H_7O_2)_3 \times H_2O$ and Na_3VO_4 concentrations were 0.02 M and 0.1 M, respectively. The solutions were then aged for 5 h in tightly closed test tubes using an oven preheated at 120 °C. After aging, the resulting dispersions containing the precipitated particles were cooled down to room temperature, centrifuged at 9000 rpm for 15 min to remove the supernatants, and washed twice with ethanol and once with Milli-Q water (i.e. adding around 10 mL of the corresponding liquid, centrifuging, and removing supernatants above the particle precipitates). The particle concentration was determined by drying 1 mL of the particle suspension in water and weighting the obtained solid residue, resulting in 1.4 mg/mL.

Purchase of Au particles

Commercial gold particles of 150 nm diameter were purchased from BBI solutions. Concentration of Au particles was taken from the web-site of BBI solutions.

4. Characterisation of as synthesized particles

Size distributions and the mean diameter of the inorganic cores of the synthesized particles (d_c) were determined by transmission electron microscopy (TEM). For this the free software ImageJ was used. To obtain TEM images, a drop of the particle solution (3 µL) was deposited on the top of a copper grid coated with a layer of carbon. TEM pictures were recorded in a Philips 200CM, and the size distribution was derived. Dynamic light scattering (DLS) and laser Doppler anemometry (LDA) analyses of the particles in water were performed with a Malvern Zetasizer Nano-ZS90. TEM images of uncoated particles and the corresponding mean values ± standard deviations of the core diameters (d_c), hydrodynamic diameters (d_h), and zeta potentials (ζ) measured a pH 7 are presented below.

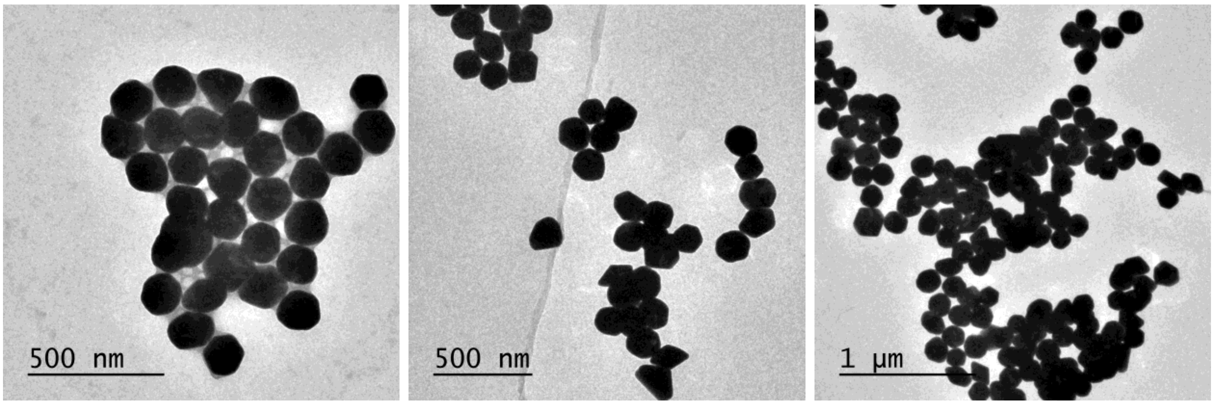

Figure S2: TEM image of Au particles, d_c = 150 ± 12 nm, d_h= 150 ± 5 nm, ζ = -30 ± 2mV.

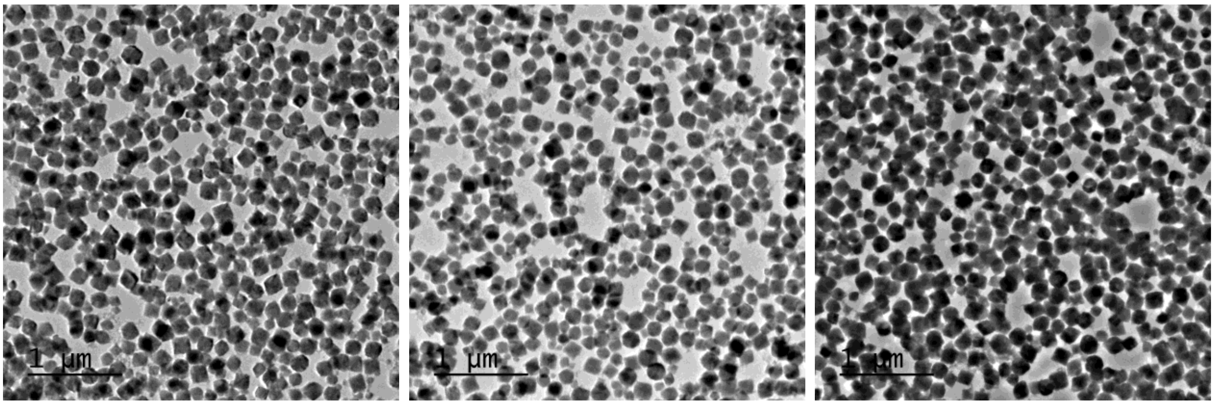

Figure S3: TEM image of YVO_4 particles, d_c = 145 ± 25 nm, d_h= 150 ± 7 nm, ζ = -40 ± 2 mV.

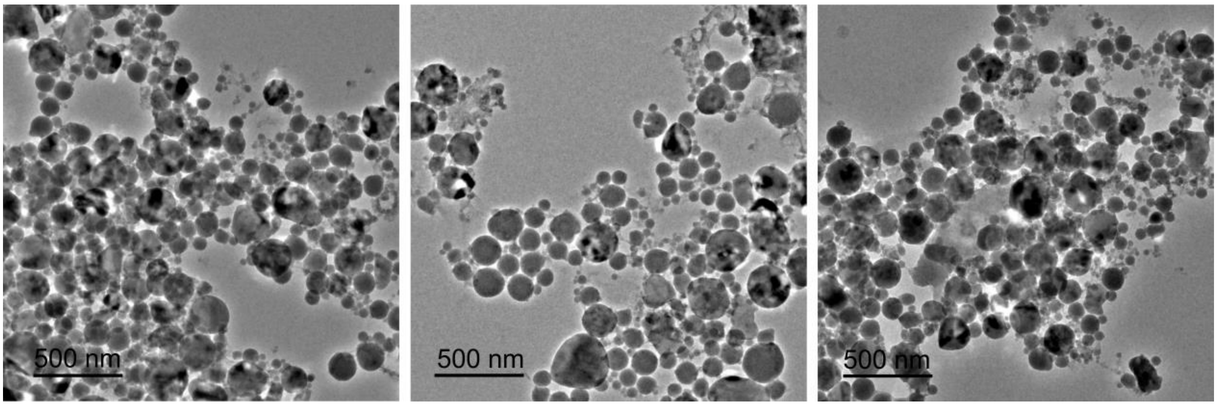

Figure S4: TEM image of Si particles, d_c = 140 ± 47 nm, d_h= 170 ± 16 nm, ζ = -45 ± 3 mV.

5. Conjugation of polymer with dye

The non-biodegradable synthetic polyelectrolyte poly(allylamine hydrochloride) (PAH) was conjugated with DY-505 dye, as follows: 15 mg of PAH were dissolved in 1 mL of 50 mM borate buffer at pH 8.5. Afterwards, 100 µL of a DY-505-NHS-ester solution in DMSO (10 mg/mL) were added, and the mixture was stirred for 90 minutes at room temperature. The final solution was purified by exclusion chromatography (PD-10 Desalting Column, GE Healthcare Life Sciences), eluted with an aqueous 0.05 M NaCl solution, and the pH was adjusted to 6.5. The final PAH-DY-505 concentration was 4 mg/mL.

6. Polymer coating of particles

Gold (Au), silicon (Si), and yttrium vanadate (YVO_4) particles were labelled with DY-505 by the following different strategies with the aim of having different distances between the particle surfaces and the dye molecules. The DY-505 dye was either deposited directly onto the particle surface by coating them with PAH conjugated with the dye (i.e. coating without spacer), or over an additional layer of polymers with different molecular weight (i.e. with a PEG spacer), which was previously deposited onto the original particle surface.

Coating of particles without spacer

Au and Si particles were coated with PAH-DY-505 as follows: 300 µL of the previously synthesised PAH-DY 505 (4 mg/mL, 0.05 M in NaCl, pH 6.5) were added to 150 µL of a water dispersion containing $5x10^9$ particles of each type (which correspond to 0.17 and 0.016 mg of Au and Si, respectively). The resulting dispersions were sonicated in water for 5 minutes and then shaken for 15 minutes at room temperature (RT). The as-coated NPs were washed by centrifugation three times with Milli-Q water (at 9000 rpm for 15 min), whereby in each step the supernatant above the particle precipitate was removed and the particles were redispersed in fresh water. In the last washing, the supernatant had no colour, indicating that the excess of reagents not attached to the particles was completely removed. The coated particles had a final architecture of particle core/PAH-DY-505. Reference particles of YVO_4 were prepared in the same way but starting from 0.5 mg of the material ($7.5x10^{10}$ particles).

Coating of particles with spacer with PEG

Au, Si and YVO_4particles were coated with polymers following a strategy based on the layer-by-layer (LbL) deposition method[6, 8]. Polymers with both positive and negative charge (PAH and PSS, respectively) were used. Moreover, in order to increase the distance between surface of particles and dye molecules, NHS-ester-PEG polymers with different molecular weights were used as additional layer, which was linked to a PAH layer through $–NH_2$ – NHS-ester bonds. For this, a first PAH polyelectrolyte layer was deposited onto the surface of the particles, as follows: 300 µL of an aqueous PAH solution (10 mg/mL, 0.05 M in NaCl, pH 6.5) were added to 500 µL of a water dispersion containing 2×10^9 particles of Au or Si (corresponding to 0.068 and 0.0066 mg of Au and Si, respectively). The resulting dispersions were sonicated for 5 minutes and then shaken for 15 minutes at room temperature. The as-coated particles were washed by centrifugation three times with Milli-

Q water and redispersed in 500 µL of water. Afterwards, a PEG spacer was introduced, as follows: 200 µL of a NHS ester-polyethileneglycol aqueous solution (11 mg/mL) with different molecular weights were added to 500 µL of the different core/PAH particle suspensions, and the mixtures were left at room temperature for 3 h with agitation. After the washing steps (centrifugation, removal of the supernatants, addition of fresh water and redispersion), particles were redispersed in 200 µL of water. Then, 300 µL of an aqueous PSS solution (10 mg/mL, 0.05 M in NaCl, pH 6.5) were added, and the dispersions were again sonicated for 5 minutes and then shaken for 15 minutes at room temperature. The particles were washed by centrifugation three times, redispersed in 200 µL of water, and finally, 300 µL of an aqueous PAH-DY-505 solution (4 mg/mL, 0.05 M in NaCl, pH 6.5) were added. The dispersions were again sonicated for 5 min, shaken for 15 min, and washed and centrifuged. In the last washing, the supernatant had no colour, indicating that the excess of reagents was completely removed. The reference YVO_4 particles were coated by following the same protocol but starting from 0.5 mg (7.5×10^{10} particles). The coated particles had a final architecture of particle core/PAH/PEG/PSS/PAH-DY-505, and were finally redispersed in 500 µL of water.

7. Basic colloidal characterization of coated particles

In total 3×5 = 15 samples were prepared, consisting of Si, Au, and YVO_4 particles with 6 different shell architectures (one particle core/PAH-DY-505 geometry and four particle core/PAH/PEG/PSS/PAH-DY-505 geometries with PEG of different molecular weight), so that different distances between the particle surface and the dye molecules were obtained.

Each step of the LbL polyelectrolyte coating and the conjugation with PEG was verified by measuring the changes in the zeta-potential of the particle suspensions. Moreover, no significant agglomeration was observed after the different coating steps, as indicated by the hydrodynamic diameters obtained by DLS (Table S1, illustrated for the sample coated with PEG 10 kDa). The final hydrodynamic diameters and zeta-potentials (measured at pH 7) of all samples obtained by DLS and LDA are presented in Table S2.

Sample	particle core		particle core /PAH		particle core /PAH/PEG		particle core /PAH/PEG/PSS		particle core /PAH/PEG/PSS /PAH-DY-505	
	ζ [mV]	d_h [nm]	ζ [mV]	d_h [nm]	ζ [mV]	d_h [nm]	ζ [mV]	d_h [nm]	ζ [mV]	d_h [nm]
Au	-30±2	150±5	+27±3	152±7	+2±2	160±9	-37±1	165±7	+26±2	177±6
Si	-45±3	170±16	+35±4	183±13	+11±2	190±19	-41±3	215±17	+18±2	185±15
YVO_4	-40±2	150±7	+31±2	168±9	+6±1	175±11	-45±1	186±10	+27±2	194±18

Table S1: Zeta-potential ζ and hydrodynamic diameter d_h after each coating step for particles made with 10 kDa PEG.

	particle core /PAH-DY-505		particle core /PAH/PEG(2kDa)/PSS /PAH-DY-505		particle core /PAH/PEG(6kDa)/PSS /PAH-DY-505		particle core /PAH/PEG(10kDa)/PSS /PAH-DY-505		particle core /PAH/PEG(20kDa)/PSS /PAH-DY-505	
	**ζ [mV]**	**d_h [nm]**	**ζ [mV]**	**d_h [nm]**	**ζ [mV]**	**d_h [nm]**	**ζ [mV]**	**d_h [nm]**	**ζ [mV]**	**d_h [nm]**
**Au**	+31±1	152±6	+18±2	162±5	+14±1	176±8	+26±2	177±6	+24±1	182±6
**Si**	+10±1	168±19	+13±2	179±15	+21±1	180±20	+18±2	185±15	+19±1	191±2
**YVO_4**	+20±2	158±6	+19±7	169±17	+39±2	175±14	+27±2	194±18	+28±2	200±16

Table S2: Hydrodynamic diameters $d_h$and zeta-potentials ζ of fabricated particles dispersed in water.

Determination of the particle concentration in the final suspensions

The concentration of the final samples was measured with ICP-MS following reported methodology.[9, 10] For the digestion of Au and YVO_4 particles, 100 µL of freshly prepared aqua regia (HCl:HNO_3 = 3:1 in volume) was added to 100 µL of the coated Au and YVO_4 particle suspensions, and the mixtures were kept in an auto-shaker overnight at room temperature. Afterwards, 1.8 mL of Milli-Q water were added (final solution volume = 2 mL, leading to a dilution factor = 20). For the digestion of Si particles, 100 µL of concentrated hydrofluoric acid (HF) were added to 100 µL of the dispersion of the coated silicon particles and kept in an auto-shaker for 48 hours. Then, 1.8 mL of 2% HNO_3 were added to the sample. Before the measurement took place, the ICP-MS setup was calibrated with a freshly prepared serial dilution of Au, Y, and Si (Roth Standard (1000 mg/mL)). The calibration curve was constructed using a concentrations series from 2 to 2500 parts per billion (ppb). Additionally the auto tuning solution from Agilent for ICP-MS 7500cs with a standard concentration of 1 µg/L of Ce, Co, Li, Mg, Tl, and Y was used to set the general background as well as to calibrate the electrical field of the lenses and the magnetic quadrupole field in strength and frequency. In the calibrated setup, the oxidation species rate was lower than 0.8% and the double charge rate was below 2%. The samples were introduced into the ICP-MS setup through a perfluoroalkoxy alkane (PFA) based microflow spray chamber, where the aqueous sample was nebulized, introduced into the argon gas flow, and transported to the torch, where it was ionized in an argon plasma of around 6000 °C. After ionization, the sample was pre-sorted using an omega lens, element-wise separated in a quadrupole field through the mass to charge rate, again sorted using kinetic barriers and a charged lens system, and finally detected with either an analog or a digital detector depending on the count rate. Date were treated using the calibration curve which usually transfer the counts per seconds (CPS) values into elemental concentrations. The raw data were then multiplied by the dilution factor to get the actual elemental concentrations of Au, Y, and Si of the samples.

The volume and weight of the different particles were calculated from their size (average diameter measured by TEM, Figures S1 to S3) and density, as follows:

Volume of an Au particle of 150 nm (radius $r_c = d_c/2 = 75$ nm):

$$V_c = \frac{4}{3}\pi {r_c}^3 = \frac{4}{3}\pi(75\,nm)^3 = 1.77 \cdot\ 10^6\ nm^3 \cdot \left(\frac{1\ cm}{10^7 nm}\right)^3 = 1.77 \cdot\ 10^{-15}\ cm^3$$

The mass of one Au particle core can be then calculated from the density of the material (ρ_{Au}=19.3 g · cm^{-3}):

$$m_c = V_c \rho_{Au} = 1.77 \cdot\ 10^{-15} cm^3 \cdot \frac{19300\ mg}{cm^3} = 34.16 \cdot 10^{-12}\ mg$$

Volume of a Si particle of 140 nm (radius r_c = 70 nm):

$$V_c = \frac{4}{3}\pi r_c{}^3 = \frac{4}{3}\pi(70\ nm)^3 = 1.77 \cdot\ 10^6\ nm^3 \cdot \left(\frac{1\ cm}{10^7 nm}\right)^3 = 1.44 \cdot\ 10^{-15}\ cm^3$$

The mass of one Si particle core can be then calculated from the density of the material (ρ_{Si} =2.33 g · cm^{-3}):

$$m_c = V_c\rho_{Si} = 1.44 \cdot\ 10^{-15} cm^3 \cdot \frac{2330\ mg}{cm^3} = 3.33 \cdot 10^{-12}\ mg$$

Volume of a YVO_4 particle of 145 nm (radius r_c = 72.5 nm):

$$V_c = \frac{4}{3}\pi r_c{}^3 = \frac{4}{3}\pi(72.5\ nm)^3 = 1.59 \cdot\ 10^6\ nm^3 \cdot \left(\frac{1\ cm}{10^7 nm}\right)^3 = 1.59 \cdot\ 10^{-15}\ cm^3$$

The mass of one YVO_4 particle core can be then calculated from the density of the material (ρ_{YVO4} =4.22 g · cm^{-3}):

$$m_c = V_c\rho_{YVO4} = 1.59 \cdot\ 10^{-15} cm^3 \cdot \frac{4220\ mg}{cm^3} = 6.71 \cdot 10^{-12}\ mg$$

The concentration of the final suspensions in number of particles per solution volute were obtained from the ICP-MS data of elemental Au, Si and Y. In the case of the reference YVO_4 particles, an additional conversion factor was used to convert Y concentration data to YVO_4 concentrations (1 mol of YVO_4 corresponds to 203.8 g of YVO_4 and contains 88.9 g of Y). The concentration calculations are illustrated for the final Au particles coated with PEG 20 kDa, which contained C'_{Au} = 26.69 ppm = 26.69 mg of Au per 1 kg of solution (ppm = parts per million). Due to the density of 1 kg/L of aqueous solution this corresponds to a mass concentration of Au of C_{Au} = 26.69 mg/L. As the mass of one Au particle is $m_c = 34.16 \cdot 10^{-12}$mg this corresponds to a particle concentration of

$$C'_{NP} = \frac{C_{Au}}{m_c} = \frac{26.69\ mg \cdot L^{-1}}{34.16 \cdot 10^{-12} mg} \approx 0.78 \cdot 10^{12} L^{-1}$$

i.e. in each litre there are $0.78 \cdot 10^{12}$ particles. Using Avogadro number $N_A = 6.02 \cdot 10^{23}$ mol^{-1} the molar concentration of Au particles is

$$C_{NP} = \frac{C'_{NP}}{N_A} = \frac{0.78 \cdot 10^{12} L^{-1}}{6.02 \cdot 10^{23} mol^{-1}} \approx 0.13 \cdot 10^{-11} mol \cdot L^{-1} = 1.3\, pM$$

i.e. 1.3 pmol Au NPs / L.

The thickness of the introduced PEG spacer layer was estimated from previous data on a different system. Figure S5 summarizes the mean PEG shell thickness around Au particles, in which PEG was directly bounded to the Au particle surface via thiol groups.[11] As observed, the thickness of the PEG shell increased with increasing the molecular weight (M_w) of PEG. The thickness of the PEG layer around the particles (take into account that they were smaller than in the present study) did not vary significantly with particle size. Thus, we assumed the same values for the bigger particles in the present study.

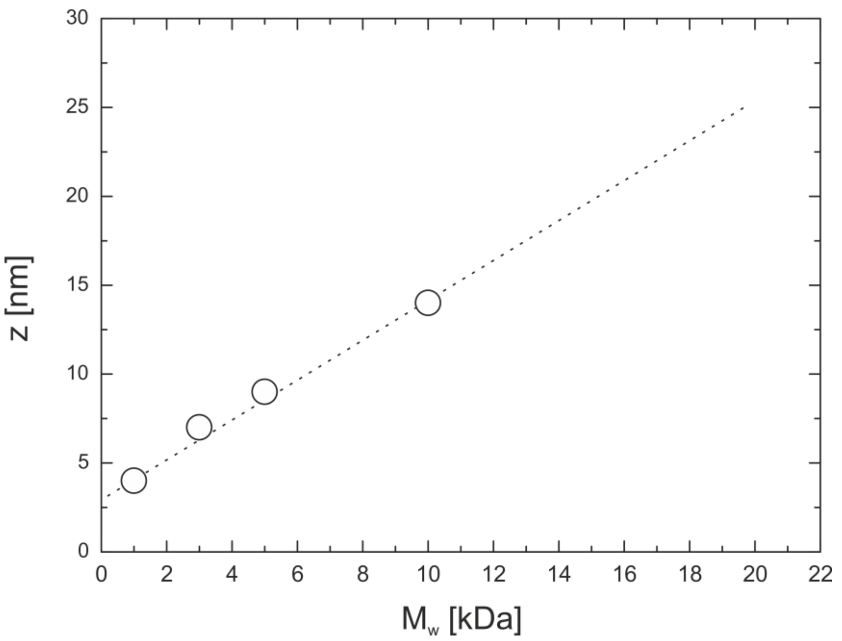

Figure S5: Estimation of the mean PEG shell thickness (z) of PEGylated Au particles in dependence of the molecular weight (M_w) of the employed PEG. These data are taken from a previous study. Adapted from.[11]

8. Photoluminescence studies

Photoluminescence spectra of the DY-505-coated particles were measured with a Fluorimeter Fluorolog-3 (JobinYvon) equipped with a Xenon Lamp. For this, 5×10^7 particles dispersed in water (the same concentration) were excited with a wavelength $\lambda_{exc} = 505\ nm$. An example is shown in Figure S6. The maximum in the PL spectrum $I_{PL}(\lambda)$ lies at a wavelength of 530 nm. The PL intensity values nm of each fluorescence spectra at 530 were normalized and combined to show their dependence on the spacer thickness) Fig. 5 of the main text).

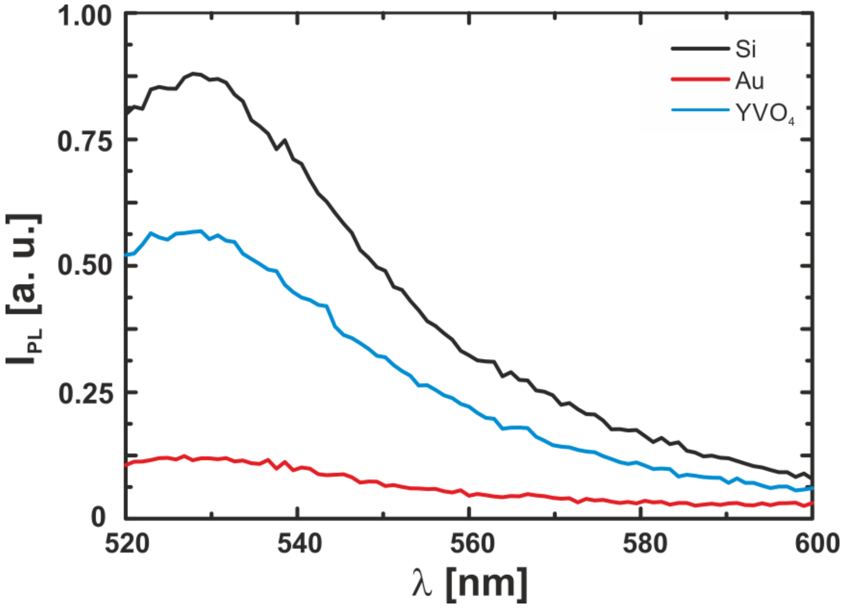

*Figure S6: Photoluminescence spectra $I_{PL}(\lambda)$ of DY-505 dye attached to Si, Au, and YVO_4 particles (in particle core/*PAH-DY-505 geometry*). The same number of particles was considered in each case.*

9. Time-resolved fluorescence microscopy

The fluorescence decay $I_{PL}(t)$of the different particles at room temperature has been investigated with a laser scanning confocal microscope MicroTime 100 (PicoQuant) equipped with a 40x objective (numerical aperture NA = 0.65) and a 70 ps pulsed diode laser (wavelength 405 nm), which implements the method of time-correlated single photon counting. The instrument response function (IRF) corresponds to approximately 100 ps. Measurements were made with a repetition pulse rate of 20 MHz and a laser power of 45 µW. The distance between the particle sample and the objective was 2 mm.

In order to measure the lifetime of PL of the differently coated particles, a drop with the prepared particle solution was placed on a cleaned cover glass and covered with another glass slice. The laser beam was focussed on the surface of the bottom cover glass and a 2D XY-scan of 50x50 μm^2 area with simultaneous time-resolved PL measurements was made. Afterwards, individual bright spots with almost similar intensity were selected (see Figure S7), assuming that these objects are individual particles. The obtained PL decay curves were fitted by a biexponential function: $I_{PL}(t) = I_0 + I_1 exp(-t/\tau_1) + I_2 exp(-t/\tau_2)$, where I_1, I_2, τ_1, τ_2,and I_0 are amplitudes, decay times, and background intensity, respectively. For a two-exponential decay an average decay time is given by: $\tau_{av} = \sum_i I_i \tau_i^2 / \sum_i I_i \tau_i$..

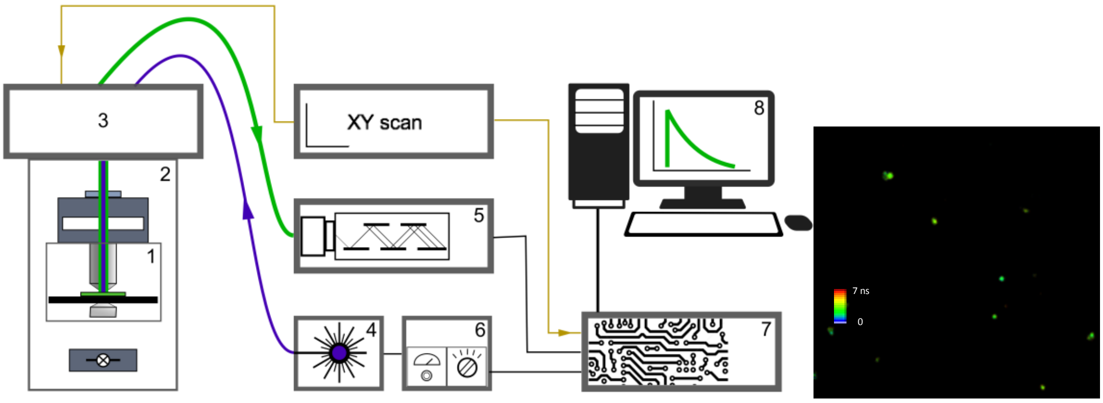

Figure S7: Left: Schematic illustration of the setup for time-resolved photoluminescence measurements (MicroTime100, PicoQuant): 1 – sample; 2 – microscope (BX-51); 3 – scanner for laser beam; 4 – diode laser emitting picosecond pulses (LDH-P-C-405-B); 5 – photodetector (PMA 185-P-M); 6 – power supply and controller of the laser (PDL 800-B); 7 - computer board for analysis of time resolved measurements (TimeHarp 200); 8 –computer with commercial software SPT-2 "SymPhoTime". Right: Typical two-dimensional (2D) XY-map of PL lifetimes from dye DY-505 coated Si particles. The color bar relates to the lifetimes of PL in the range of 0-7 ns.

10. Cell studies

To illustrate the capabilities of the here synthesized particles for bioimaging, standard particle uptake-experiments by HeLa cells were performed.

Human cervical carcinoma (HeLa) cells were obtained from the American Type Culture Collection (ATCC). HeLa cells were seeded into 8-well plates at an amount of 15.000 cells per well with 250 µL medium per well and were grown overnight in Dublecco's Modified Eagles Medium supplemented with 10% FBS and 1% P/S at 37 °C in 5% CO_2. The next day, cells were washed with PBS, and either Si-PAH-DY-505 particles or Au-PAH-DY-505 particles were added to the cells at an amount of 20 added particles per seeded cell. The HeLa cells were incubated with the particles for 24 hours. After incubation, the cell membrane and the nuclei of cells were stained with Cell Mask (1 µg/mL) and Hoechst 33342 (1 µg/mL), respectively, at 37 °C for 15 min. Cells were then washed 1 time with PBS and fresh growth medium was added to the cells.

Cells were studied with a confocal microscope (Zeiss LSM 510 Meta, with the following objective: Plan-Apochromat 63 /1.40 Oil DIC M27). An argon (Ar) laser at 488 nm and a bandpass filter BP 505-550 were used as excitation source and emission filter, respectively, to detect the DY-505-labelled particles. To image the cell membranes a HeNe laser at 543 nm was used as excitation source with a bandpass emission filter BP 560-615. To detect the cell nuclei a diode laser at 405 nm was used with a bandpass emission filter BP 420-480.Fluorescence micrographs of cells with internalized particles are shown in Figure S8.Acquisition times and excitation powers were similar for both samples. The measurements show that particles were internalized by cells, as to be expected.[12] The DY-505 dye on individual Si particles without any spacer had much higher fluorescence intensity, whereas the DY-505 dye on Au particles was almost not visible. These results confirm the statement that silicon particles less quench the dye and thus there is no need for a spacer between the surface of particles and the attached dye molecules. Thus, Si NPs are good candidates for bioimaging applications.[13,14]

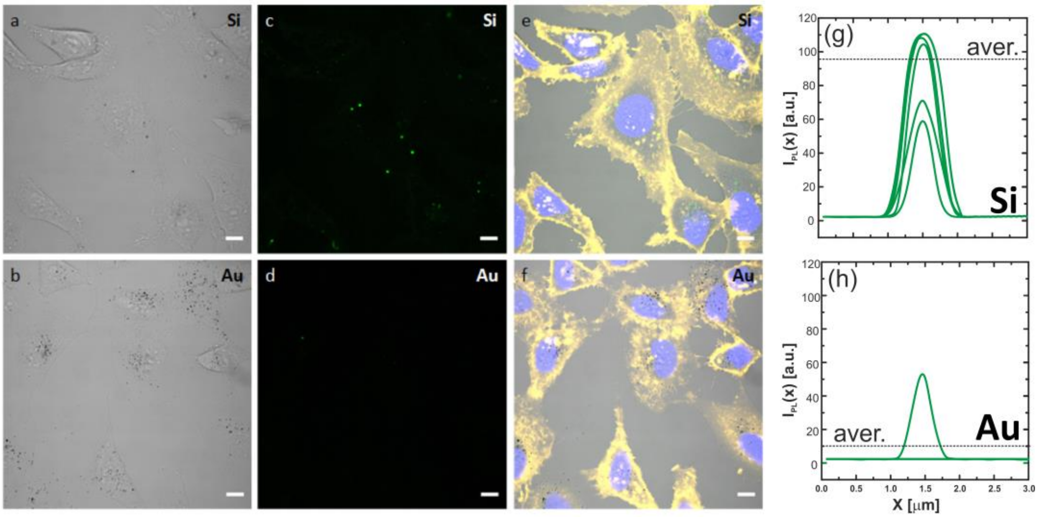

Figure S8: Optical images in transmission mode of (a, c, e) Si-PAH-DY-505, and (b, d, f) Au-PAH-DY-505 particles internalized by HeLa cells. (c, d) DY-505 fluorescence image to visualize the labelled particles. (e, f) Merged fluorescence image, in yellow the membranes, and in blue the nuclei of cells are shown.(g, h) PL intensities line scans $I_{PL}(x)$ of individual Si (g) and Au (h) particles as taken from images (c) and (d), respectively. Averaging of PL intensities over presented spectra. The scale bars correspond to 10 μm.

11. References

1. Evlyukhin, A.; Reinhardt, C.; Seidel, A.; Luk'yanchuk, B.; Chichkov, B., Optical Response Features of Si-Nanoparticle Arrays. *Phys. Rew. B* **2010,** 82, 045404.

2. Bohren, C.; Huffman, D., Absorption and scattering of light by small particles. WILEY-VCH Verlag GmbH & Co. KGaA, 1998.

3. Bharadwaj, P.; Deutsch, B.; Novotny, L., Optical Antennas. *Adv. Opt. Photonics* **2009,** 1, 438-483.

4. Novotny, L.; Hecht, B., Principles of Nano-Optics. Cambridge: Cambridge University Press, 2012.

5. Zhang, D.; Goekce, B.; Barcikowski, S., Laser Synthesis and Processing of Colloids: Fundamentals and Applications. *Chem. Rev.* **2017,** 117, 3990-4103.

6. Escudero, A.; Carrillo-Carrion, C.; Zyuzin, M.; Ashraf, S.; Hartmann, R.; Nunez, N.; Ocana, M.; Parak, W., Synthesis and Functionalization of Monodisperse Near-ultraviolet and Visible Excitable Multifunctional Eu3+, Bi3+: REVO4 Nanophosphors for Bioimaging and Biosensing Applications. *Nanoscale* **2016,** 8, 12221-12236.

7. Escudero, A.; Carrillo-Carrion, C.; Zyuzin, M.; Parak, W., Luminescent Rare-Earth-Based Nanoparticles: A Summarized Overview of their Synthesis, Functionalization, and Applications. *Top. Curr. Chem.* **2016,** 374.

8. Zyuzin, M.; Honold, T.; Carregal-Romero, S.; Kantner, K.; Karg, M.; Parak, W., Influence of Temperature on the Colloidal Stability of Polymer-Coated Gold Nanoparticles in Cell Culture Media. *Small* **2016,** 12, 1723-1731.

9. Huhn, J.; Carrillo-Carrion, C.; Soliman, M.; Pfeiffer, C.; Valdeperez, D.; Masood, A.; Chakraborty, I.; Zhu, L.; Gallego, M.; Yue, Z.; Carril, M.; Feliu, N.; Escudero, A.; Alkilany, A.; Pelaz, B.; del Pino, P.; Parak, W., Selected Standard Protocols for the Synthesis, Phase Transfer, and Characterization of Inorganic Colloidal Nanoparticles. *Chem. Mater.* **2017,** 29, 399-461.

10. Kreyling, W.; Abdelmonem, A.; Ali, Z.; Alves, F.; Geiser, M.; Haberl, N.; Hartmann, R.; Hirn, S.; de Aberasturi, D.; Kantner, K.; Khadem-Saba, G.; Montenegro, J.; Rejman, J.; Rojo, T.; de Larramendi, I.; Ufartes, R.; Wenk, A.; Parak, W., In Vivo Integrity of Polymer-Coated Gold Nanoparticles. *Nat. Nanotechnol.***2015,** 10, 619-623.

11. del Pino, P.; Yang, F.; Pelaz, B.; Zhang, Q.; Kantner, K.; Hartmann, R.; de Baroja, N.; Gallego, M.; Moller, M.; Manshian, B.; Soenen, S.; Riedel, R.; Hampp, N.; Parak, W. Basic Physicochemical Properties of Polyethylene Glycol Coated Gold Nanoparticles that Determine Their Interaction with Cells. *Angew. Chem., Int. Ed.* **2016,** 55, 5483-5487.

12. Nazarenus, M.; Zhang, Q.; Soliman, M. G.; Del Pino, P.; Pelaz, B.; Carregal-Romero, S.; Rejman, J.; Rothen-Rutishauser, B.; Clift, M. J.; Zellner, R.; Nienhaus, G. U.; Delehanty, J. B.; Medintz,

I. L.; Parak, W. J., In vitro Interaction of Colloidal Nanoparticles with Mammalian Cells: What Have We Learned Thus Far? *Beilstein J. Nanotechnol.* **2014,** 5, 1477-1490.

13. Peng, Fei; Su, Yuanyuan; Zhong, Yiling; Fan, Chunhai; Lee, Shuit-Tong; He Yao, Silicon Nanomaterials Platform for Bioimaging, Biosensing, and Cancer Therapy, *Acc. Chem. Res.*, **2014**, 47, 612–623.

14. Su, Yuanyuan; Ji, Xiaoyuan; He, Yao, Water-Dispersible Fluorescent Silicon Nanoparticles and their Optical Applications, *Adv. Mater.* **2016**, 28, 10567–10574.
